# Modeling the Effect of Environmentally Sustainable Food Swaps on Nutrient Intake in Pregnant Women

**DOI:** 10.3390/nu13103355

**Published:** 2021-09-24

**Authors:** Tian Wang, Allison Grech, Hasthi U. Dissanayake, Sinead Boylan, Michael R. Skilton

**Affiliations:** 1 Boden Collaboration for Obesity, Nutrition, Exercise and Eating Disorders, The University of Sydney, Sydney, NSW 2006, Australia; tian.wang@sydney.edu.au; 2 Sydney Medical School, The University of Sydney, Sydney, NSW 2006, Australia; agre2034@uni.sydney.edu.au (A.G.); hasthi.dissanayake@sydney.edu.au (H.U.D.); sinead.boylan@sydney.edu.au (S.B.); 3 School of Life and Environmental Sciences, The University of Sydney, Sydney, NSW 2006, Australia; 4 Sydney Institute for Women, Children and Their Families, Sydney Local Health District, Camperdown, NSW 2050, Australia

**Keywords:** nutrition, sustainability, pregnancy, nutritional requirements, food production system, environment, diet

## Abstract

Food production greatly contributes to greenhouse gas emissions (GHG), but there remain concerns that consuming environmentally sustainable foods can increase the likelihood of nutritional deficiencies during pregnancy. We identified commonly consumed foods of pregnant women and determined the effect of their replacement with environmentally sustainable alternatives on nutrient intake and measures of environmental sustainability. Dietary intake data from 171 pregnant women was assessed and foods that contributed the most to energy and protein intake were identified. Of these, foods producing the highest GHG emissions were matched with proposed environmentally sustainable alternatives, and their impact on nutrient provision determined. Meats, grains, and dairy products were identified as important sources of energy and protein. With the highest GHG emissions, beef was selected as the reference food. Proposed alternatives included chicken, eggs, fish, tofu, legumes, and nuts. The most pronounced reductions in CO_2_ emissions were from replacing beef with tofu, legumes, and nuts. Replacing one serve per week of beef with an isocaloric serve of firm tofu during pregnancy could reduce GHG emissions by 372 kg CO_2_ eq and increase folate (+28.1 µg/serve) and fiber (+3.3 g/serve) intake without compromising iron (+1.1 mg/serve) intake. Small dietary substitutions with environmentally sustainable alternatives can substantially reduce environmental impact without compromising nutrient adequacy.

## 1. Introduction

The food production system is a major contributor to global warming and environmental change, through greenhouse gas (GHG) emissions, freshwater use, land use, acidification, and eutrophication [1]. According to the EAT-Lancet Commission and the Food and Agricultural Organization, there is an urgent need to shift to environmentally sustainable diets on a global scale, by moving towards greater consumption of plant-based foods and reducing the production of less environmentally sustainable animal-derived products [1,2].

Requirements for certain nutrients are elevated during pregnancy to support maternal needs and optimize fetal growth and development [1,3]. Animal-derived foods are an important source of some of these nutrients, such as iron and zinc, and replacement with more environmentally sustainable plant-based alternatives may negatively impact the intake of these nutrients [4]. Health messaging aimed at pregnant women emphasizes the importance of animal-derived foods, particularly red meat, to meet the nutritional requirements of pregnancy [5]. While women consuming a vegetarian or vegan diet are considered to be at higher risk of iron deficiency during pregnancy [6,7], appropriately planned vegetarian diets are nutritionally adequate and suitable for pregnant women to consume [8]. However, it is not clear whether replacement of a small portion of animal-derived foods (e.g., one serve/week) with plant-based alternatives without other dietary modifications will meaningfully affect nutrient intakes among pregnant women consuming a mixed diet, who make up the majority of pregnant women in Western countries [9,10]. Accordingly, we sought to model the net nutritional and environmental effects of partial replacement of commonly consumed animal-derived foods with more environmentally sustainable alternatives within the context of a mixed diet during pregnancy.

## 2. Materials and Methods

### 2.1. Maternal Demographics and Identification of Commonly Consumed Foods

A total of 224 mothers and their babies were recruited between April 2015 and September 2016 at the Royal Prince Alfred Hospital in Sydney, Australia, from the Newborn Body Fatness study [11]. Eligibility criteria included: gestational age ≥ 34 weeks; singleton born at the Royal Prince Alfred Hospital; and completed assessment of infants’ body fatness within 24 h of birth using air-displacement plethysmography. Infants who required respiratory support or had major congenital abnormalities were excluded. Pregnant women with diabetes and preeclampsia were excluded from this dietary analysis as they would have received medical nutritional advice to manage these conditions, leaving 171 women for inclusion in this analysis (Figure S1).

Dietary intake data of pregnant women were collected using a validated food frequency questionnaire (FFQ; Dietary Questionnaire for Epidemiological Studies, version 2) [12]. The median intake per day of 96 food items was recorded and categorized according to the same grouping as Poore et al. [13]. For example, full-cream milk, reduced-fat milk, skim-milk, and flavored-milk drinks were combined into the “milk” food group. For each food group, the energy and protein content of individual food items were analyzed using the AUSNUT 2011-13 AHS Food Nutrient Database [14] (Table S1). Food groups with more than one food item were calculated based on the median intake of each food items. Food items were then ranked by energy and protein intake.

Ethics approval was granted by the Sydney Local Health District (HREC/14/RPAH/478), with written informed consent provided by the participating mothers.

### 2.2. Environmentally Sustainable Food Alternatives

The commonly consumed foods items, those with a relatively high mean GHG emissions per kilogram (kg) retail weight, based on data from Poore et al. [13], were selected as reference foods. These include beef, chicken, white fish, and milk. Foods with lower mean GHG emissions per kg retail weight were proposed as more environmentally sustainable alternatives. These include beans and legumes, tofu, and mixed nuts. Using the Australian Guide to Healthy Eating [15] as a basis, the standard serve size of reference foods (cooked-size) was determined, and the energy and protein content were calculated using the AUSNUT 2011-13 AHS Food Nutrient Database [14]. The portion size of the proposed food substitutes was determined by matching the energy and protein content of reference foods.

Nutrients included in the analysis were protein, calcium, iron, zinc, iodine, folic acid, saturated fat, and dietary fiber on the basis of these being essential nutrients during pregnancy according to the National Health and Medical Research Council [16] and the American College of Obstetricians and Gynecologists [17], and/or associated with non-communicable disease risk. Estimated absorbed iron was calculated based on the following assumptions: 100% of iron in plant-based foods was non-heme iron; 60% of iron in animal-derived foods was non-heme and 40% was heme iron [18]; 16.8% of non-heme iron and 25% of heme iron are absorbed within the context of a mixed diet [19].

Mean global measures of environmental sustainability, specifically GHG emissions (kg CO_2_ eq), land use (m^2^ by years occupied), acidifying emissions (g SO_2_ eq), eutrophying emissions (g PO_4_^3−^ eq), and stress-weighted water use (L) of the food production, were derived from Poore and Nemecek [13]. The total GHG emissions (kg CO_2_ eq), land use (m^2^), acidifying emissions (g SO_2_ eq), eutrophying emissions (g PO_4_^3−^ eq), and stress-weighted water use (L) of consuming one serve of an individual food item per week for the entire duration of pregnancy (270 days) were calculated. GHG emissions of an average passenger vehicle were derived from the United States Environmental Protection Agency GHG equivalencies calculator [20].

Furthermore, we modeled the effect on nutrient reference values (NRVs) of replacing one serve of beef with an isoenergetic serve of firm tofu in this population. The NRVs of important nutrients (protein, dietary fiber, iron, zinc, calcium, and folate) during pregnancy were acquired from the National Health and Medical Research Council [16] and the number (%) of people meeting NRVs before and after this replacement were calculated.

### 2.3. Statistical Analyses

Data are presented as mean for continuous variables and proportions for categorical variables, unless otherwise noted. Histograms were generated for visual assessment of normality. Statistical analyses were performed with IBM^®^ SPSS Statistics 26 Software (IBM, Armonk, NY, USA).

## 3. Results

### 3.1. Participant Dietary Characteristics: Nutrition and Commonly Consumed Foods

A total of 171 pregnant women without diabetes and/or preeclampsia were included in the dietary intake analysis. Their mean age was 33.5 ± 4.5 years. In total, 22% of them had pre-pregnancy BMI ≥ 25 kg/m^2^, classified as having overweight or obesity.

Table 1 lists the food items that were commonly consumed and contributed the most to pregnant women’s energy and protein intake. Rice, pasta, full-cream milk, yogurt, chicken, beef, and mixed dishes with cereal as the major ingredient were important sources of both energy and protein. The dietary intake analysis can be found in Table S1.

### 3.2. Environmentally Sustainable Food Alternatives

From the identified commonly consumed foods, four food items with relatively high GHG emissions were selected as reference foods: beef (99.5 kg CO_2_ eq per 1 kg), chicken (9.9 kg CO_2_ eq per 1kg), white fish (13.6 kg CO_2_ eq per 1 kg), and milk (3.2 kg CO_2_ eq per 1 L) [12]. Food items with lower GHG emissions than each reference food, including the other reference foods, were proposed as more environmentally sustainable alternatives. Beef was selected as the primary reference food given its contributions to average energy intake, protein intake, and GHG emissions, and its proposed more environmentally sustainable alternatives include other red meats (pork and lamb), chicken, egg, fish, beans and legumes, tofu, and nuts. The proposed more environmentally sustainable alternatives for other reference foods could be found in the Supplementary Material (Tables S2–S10).

#### 3.2.1. Replacement of Less Environmentally Sustainable Foods with Energy-Matched Serves of More Environmentally Sustainable Alternatives

The nutrient analysis and measures of environmental sustainability for the beef and isoenergetic serves of more environmentally sustainable alternatives are shown in Table 2 and Table 3. The net difference in nutrients and measures of environmental sustainability of replacing one serve of a reference food for an isoenergetic serve of a more environmentally sustainable alternative are shown in Table S2 (beef), Table S3 (chicken), Table S4 (white fish), and Table S5 (milk). In general, animal-derived alternatives have a similar weight (mean cooked weight: 69 g) to beef (65 g cooked weight) whereas an isoenergetic serve of a plant-based alternative (except nuts) weighs approximately 1.5 to 2 times as much. Protein content was generally higher in animal-derived options than isoenergetic serves of plant-based alternatives, with the exception of nuts. Animal-derived foods were important sources of iodine, particularly eggs and white fish. Plant-based foods contain fiber and were generally rich sources of calcium and folate compared to animal-derived foods.

Not accounting for bioavailability, the iron content provided by one serve of beef is 1.7 mg (Table 2) and, of the food items analyzed, was the richest animal-derived iron source. The overall iron content of plant-based alternatives was high, with mixed beans, lentils, and tofu (firm and silken) being the richest sources. The estimated absorbed iron from isoenergetic serves of mixed beans, lentils, and tofu (firm and silken) was slightly higher than from beef.

Using data from Poore et al. [13], the production of beef produced far greater GHG emissions (10.0 kg CO_2_ eq per serve) than other animal-derived alternatives and plant-based alternatives (Table 3). Extrapolating these results, if pregnant women replace one serve of beef with one isoenergetic serve of firm tofu each week throughout their pregnancy, GHG emissions would be reduced by 372.2 kg CO_2_ eq, equivalent to the emissions produced by a typical passenger vehicle driven for 1498 km (Table S2). For other measures of environmental sustainability, all food alternatives have land use less than 2.1 m^2^, except for lamb (25.4 m^2^), per isoenergetic serve (Table 3). Most food alternatives have acidifying emissions of lower than 10 g SO_2_ eq per isoenergetic serve. White fish produced the highest eutrophying emissions (24.1 g PO_4_^3−^ eq). Lastly, pork, lamb, and white fish have higher stress-weighted water use (L) than beef (3473.3 L) per serve. Modeling of this replacement of one serve of beef with one isoenergetic serve of firm tofu indicates a small positive impact or no impact on nutrient intakes (Table 4). For example, it would not meaningfully impact iron intake (+1.1 mg/serve), whilst folate (+28.1 µg/serve) and dietary fiber (+3.3 g/serve) would both increase. The exception is zinc, which decreases by 0.5 mg per serve. The net results of this substitution would be that among women who consume a mixed diet, the proportion who meet NRVs for zinc fall by 11%, and the proportion of pregnant women meeting NRVs for calcium and fiber increase by 5% and 2%, respectively.

Further analyses of replacing reference foods with environmentally sustainable alternatives can be found in the Supplementary Material. For example, replacing one serve of beef with one isoenergetic serve of mixed beans reduces protein, saturated fat, and zinc content as expected, while increasing dietary fiber, iron, calcium, and folate content, as well as lowering all measures of environmental sustainability (Table S2). In addition, replacing one serve of milk with an isoenergetic serve of soy milk does not negatively impact on calcium content (+68.4 mg/serve) but reduces the impact on all measures of environmental sustainability (Table S5). The net effect of this replacement per day over the course of an entire pregnancy would reduce GHG emissions by 138.9 kg CO_2_ eq, equivalent to the emissions produced by a typical passenger vehicle driven for 559 km (Table S5).

#### 3.2.2. Protein-Matching Environmentally Sustainable Alternatives

The nutrient analysis and measures of environmental sustainability for the reference foods and protein-matched serves of more environmentally sustainable alternatives are shown in Table S6. The net differences in nutrients and measures of environmental sustainability of replacing one serve of a reference food for a protein-matched serve of a more environmentally sustainable alternative are shown in Table S7 (beef), Table S8 (chicken), Table S9 (white fish), and Table S10 (milk).

The weight of protein-matched portion sizes for all of the more environmentally sustainable alternatives was markedly greater than that of beef, with some plant-based alternatives weighing more than four times as much as one serve of beef (Table S4). Similar to the isoenergetic serves, animal-derived foods were rich in iodine, whilst plant-based alternatives were rich sources of calcium, folate, and dietary fiber.

In general, plant-based alternatives were high in iron when matching protein content, with the richest sources from silken tofu, mixed beans, and lentils (Table S4). The estimated iron absorption of these three foods was three times as much as beef in one protein-matched serve. The average zinc content provided by plant-based alternatives was lower than that of beef but higher than other animal-derived alternatives.

Using protein-matched serves did not markedly alter the results regarding the net benefits to environmental sustainability, particularly GHG emissions. For example, if pregnant women substitute one serve of beef with a protein-matched serve of firm tofu each week throughout the course of pregnancy, GHG emissions would be reduced by 363 kg CO_2_ eq, equivalent to 1461 km of typical driving distance by an average passenger vehicle. The net results of this replacement would decrease the proportion of women who meet NRVs for zinc by 5% and increase the proportion of pregnant women meeting NRVs for calcium and fiber by 10% and 2%, respectively (Table 5). For other measures of environmental sustainability, all food alternatives have land use less than 6.4 m^2^, except for lamb (36.3 m^2^), per protein-matched serve (Table S6). Most food alternatives have acidifying emissions of lower than 15 g SO_2_ eq per protein-matched serve. White fish produced the highest eutrophying emissions (20.8 g PO_4_^3−^ eq). Lastly, protein-matched serves of more environmentally sustainable alternatives have relatively high stress-weighted water use on average, although this varied greatly, ranging from 861L per serve for firm tofu through to over 13,000 L per serve for mixed nuts and lamb.

## 4. Discussion

The Food and Agriculture Organization of the United Nations and others has identified an urgent need to shift to environmentally sustainable diets on a global scale [1,2]. Concerns over nutrient adequacy in populations with high nutrient demands, such as pregnant women, are potential challenges to the broad implementation of environmentally sustainable diets. Our findings indicate these concerns are likely misplaced within the context of a mixed diet. Modeled replacement of animal-derived food with more environmentally sustainable plant-based alternatives has only a small effect on overall nutrient intake but a considerable positive effect on environmental sustainability.

There remain concerns among many practitioners and community members regarding the potential risk of nutrition inadequacy of pregnant women consuming plant-based diets [21,22]. Our focus was not plant-based diets but rather environmentally sustainable foods within the context of mixed diets. Focusing on mixed diets enables our findings to be relevant to a large proportion of the population for whom consuming a purely vegetarian or plant-based diet is neither practicable nor desirable [9,10].

In general, our results support animal-derived foods as a rich source of zinc, and plant-based foods as being rich in calcium, folate, and dietary fiber. A specific swap replacing one serve per week of beef with firm tofu reduces zinc and protein levels, while calcium, folate, and dietary fiber increases. The absolute differences in the nutrient intake of this swap were small. In this modeling, the largest differences were for calcium (raised by about 13% of a standard deviation), zinc (reduced by about 10% of a standard deviation), and fiber (raised by about 5% of a standard deviation), resulting in an increase in the proportion of pregnant women who meet NRVs of calcium, dietary fiber, and iron, but a decrease for zinc. Maternal zinc deficiency during pregnancy may increase the risk of low birth weight and small for gestational age infants [23], although severe zinc deficiency is rare. Indeed, in our population, the majority of women met the NRVs for zinc, based on both actual and modeled intakes. Furthermore, zinc is a common ingredient in pregnancy multivitamins, which are used by approximately 70–80% of women in the USA, Europe, and Australia [7,24,25,26]. The amount of zinc in such multivitamins (typically 11 mg per day) [27] exceeds the NRV for zinc.

Alongside folate, the public is perhaps most aware of concerns regarding sufficient iron intake during pregnancy [3,7]. Plant-based foods are a good source of overall dietary iron, but this does not account for differences in the bioavailability of heme and non-heme iron. Heme iron is only found in animal-derived meat products. Heme iron constitutes approximately 40% of total iron from animal-derived meat products and it is more readily absorbed by humans than non-heme iron [3]. To account for this, we estimated the amount of absorbed iron. This estimation did not account for the increased absorption of non-heme iron during pregnancy [28], and as such is a conservative estimate of absorbed iron from plant-based foods. Nonetheless, both total dietary iron intake and estimated absorbed iron were slightly higher after replacing a serve of beef with an isoenergetic serve of firm tofu (Table S2), although the magnitude did not appear to be clinically meaningful on an individual basis. It is notable that the proportion of women meeting NRVs for iron intake by diet alone was low in our population (about 5%). This is consistent with other studies of pregnant women in Australia [7], and with dietary modeling undertaken as part of the development of the Australian Guide to Healthy Eating, in which no dietary models could provide sufficient iron to meet the needs of pregnant women [29].

The low prevalence of participants meeting the NRVs for folate, iron, and fiber, for both actual and modeled intakes, highlights the necessity of appropriately planned diets, by health professionals, such as dietitians or individuals with nutrition training, to fulfill the nutritional needs of women during pregnancy. The use of dietary supplements during pregnancy may at least partially alleviate these deficiencies, irrespective of the background diet. There is currently limited publicly available information concerning the environmental sustainability of pregnancy supplements.

Iron and zinc absorption can be affected by other factors. Within the context of a mixed diet, non-heme iron and zinc absorption can be enhanced by other components of the diet, including meat, poultry, fish, and other seafood [30,31], alongside vitamin C-rich foods, e.g., citrus fruits and green leafy vegetables [32]. Therefore, one way to implement a one-serve per week replacement within the context of a mixed diet whilst maintaining the effect of iron and zinc absorption enhancers (e.g., meat products, green leafy vegetables) would be to replace half the portion of a less environmentally sustainable animal-derived meat product with a more sustainable plant-based food twice per week.

The most environmentally sustainable alternatives produced approximately 98% less GHG emissions than one serve of beef when matched for energy or protein content. To facilitate a broader understanding of the impact of incorporating more environmentally sustainable foods, we compared GHG emissions generated in food production to those produced by typical passenger vehicle usage. Using the example above, replacing one serve of beef with an isoenergetic serve of firm tofu per week during pregnancy could reduce GHG emissions by the equivalent to those produced by a typical passenger vehicle driven for 1498 km. Similarly, most of the proposed environmentally sustainable alternatives have a lesser environmental impact when assessed by other measures of environmental sustainability (including land use, acidifying emissions, and eutrophying emissions), although water use in the production of some plant-based alternatives (e.g., legumes and beans) appears to be similar to that of some animal-derived foods.

When matching the energy and protein content to reference foods, the portion size of plant-based alternatives is two to four times heavier than animal-derived foods, consistent with the density of energy and protein being notably higher in animal-derived foods. Plant-based alternatives are rich in dietary fiber with lower energy density, which increase satiety, helping to maintain a healthy weight by limiting calorie intake [33,34], and optimize weight gain during pregnancy [35,36]. Excessive protein intake from animal sources, primarily meat products, may also increase the risk of overweight and obesity in offspring [37].

Our study has a number of limitations. We used an FFQ administered in the immediate postpartum period. Women were asked to recall their habitual diet during pregnancy, which we validated against dietary biomarkers in a subgroup [38]. FFQs are well described as tools for assessing habitual diet over 6–12-month periods, although we cannot rule out that there may have been a greater emphasis on third trimester intake due to recency bias. Furthermore, detailed information of dietary intake (e.g., ingredients of mixed dishes) is difficult to assess but will include meat or other food items as an ingredient. As such, the values for meat and other food items will have been underestimated. Nonetheless, previous research has shown dietary patterns during pregnancy remain relatively stable when compared to pre-pregnancy intake [39,40,41] and are not significantly different to those of non-pregnant women of reproductive age [42]. Our study population from which we identified the commonly consumed food items during pregnancy has a relatively low prevalence of overweight and obesity (22%). This is less than in the general population in Western countries, where up to 50% of women have overweight or obesity before pregnancy [43,44], and is likely due at least in part to the exclusion of women with diabetes and preeclampsia from our analyses. Future research may seek to determine the environmental sustainability of foods consumed by representative samples of pregnant women. We mainly focused on GHG emissions given their contribution to global warming and did not describe the impact of environmentally sustainable foods on the economy and society. A range of indicators of economic and societal aspects [45] (e.g., affordability, employment, and food insecurity) can be used to assess the effects of improving environmental sustainability and should be the topic of future research. We acquired measures of environmental sustainability from a global dataset by Poore and Nemecek [13], consisting of data derived from 570 studies in 119 countries to ensure that our findings can be broadly generalizable. Future studies could employ country-specific measures of environmental sustainability to enable a more geographically accurate indication of the environmental impact of these food swaps. The role of food–food interactions that influence absorption was beyond the scope of our current study; however, future research should look to model these interactions within the context of dietary changes to promote environmental sustainability. Finally, to translate our findings into practice, the acceptability and popularity of proposed environmentally sustainable options need to be taken into consideration. Future research should identify whether there are unique challenges or opportunities for promoting environmentally sustainable foods during pregnancy. Nutrition communicators, dietitians, and practitioners may need to focus on the promotion of health benefits of environmentally sustainable plant-based foods, and provide practical advice (e.g., design recipes) in incorporating these replacements into their individual diets.

## 5. Conclusions

Our research highlights simple dietary substitutions that can substantially reduce environmental impact without compromising essential nutrient intake during pregnancy. Moving forward, environmentally sustainable food replacements should be the focus of applied clinical research and inform nutrition practice and policy development.

## Figures and Tables

**Table 1:** Commonly consumed food items that contributed most to the energy and protein intake of pregnant women.

Food Items	Energy (kJ/Day)	Food Items	Protein (g/Day)
Rice	539	Beef	6.4
Pasta	518	Chicken	4.6
Full-cream milk	281	Pasta	4.5
Chocolate	271	Yogurt	3.9
Yogurt	236	Full-cream milk	3.4
Chicken	199	Rice	2.3
Cakes	148	Fish	2.2
Beef	142	Mixed dishes *	1.9
Mixed dishes *	131	Lamb	1.9
Tropical fruits	123	Eggs	1.6

* Mixed dishes with cereal as the major ingredient.

**Table 2:** Nutrient analysis of beef and isoenergetic serves of more environmentally sustainable options ^#^.

Foods	Serve Size, ^†^ g	Protein, g	SFA, g	Dietary Fiber, g	Fe, mg	Estimated Fe Absorption, mg	Zn, mg	Ca, mg	Iodine, µg	Folate, * µg
Beef	65	20.2	1.2	0.0	1.7	0.33	5.1	5.2	1.1	0.0
Pork	83	23.7	0.6	0.0	0.8	0.16	2.0	3.3	0.7	36.6
Lamb	49	14.2	2.5	0.0	1.1	0.22	2.1	4.5	0.2	7.9
Chicken	74	21.4	0.9	0.0	0.4	0.07	0.6	6.7	0.4	2.2
Egg	81	10.0	2.1	0.0	1.3	0.26	1.0	31.5	38.2	67.0
Salmon	39	11.4	1.6	0.0	0.6	0.11	0.2	3.9	3.8	0.0
White fish	89	23.4	0.5	0.0	0.5	0.10	0.6	38.3	39.6	1.8
Beans, mixed	110	7.1	0.1	6.8	2.2	0.38	0.9	47.5	0.6	74.0
Chickpeas	101	6.4	0.2	4.7	1.8	0.31	1.0	45.4	0.5	63.6
Lentils	133	9.0	0.1	4.9	2.7	0.45	1.2	22.6	0.7	26.6
Baked beans	133	6.5	0.1	6.9	1.3	0.23	0.7	51.7	2.0	66.3
Tofu, firm	94	11.3	0.9	3.3	2.7	0.46	1.6	300.0	2.7	28.1
Tofu, silken	210	11.3	0.7	4.8	3.8	0.64	1.1	50.4	2.5	27.3
Mixed nuts	18	3.9	1.4	1.1	0.5	0.09	0.7	16.2	0.1	10.2

^#^ Energy content of foods was matched to one serve of beef (471 kJ). ^†^ Cooked weight. * Dietary folate equivalents. Ca, calcium; Fe, iron; SFA, saturated fatty acid; Zn, zinc.

**Table 3:** Environmental sustainability of beef and isoenergetic serves of more environmentally sustainable options ^#^.

	Estimated Effect of Consuming One Serve					Overall Estimated Effect of Consuming One Serve per Week for Duration of Pregnancy					
Foods	GHG Emissions, kg CO_2_ eq	Land Use, m^2^	Acid., g SO_2_ eq	Eutroph., g PO_4_^3−^ eq	Stress-Weighted Water Use, L	kg CO_2_ eq	Equiv km ^	Land Use, m^2^	Acid., g SO_2_ eq	Eutroph., g PO_4_^3−^ eq	Stress-Weighted Water Use, L
Beef	10.0	32.6	31.9	30.1	3473	383.8	1545	1258.2	1229.7	1162.5	133,968.2
Pork	1.4	2.0	16.5	8.8	7722	54.8	221	77.5	635.6	340.3	297,842.7
Lamb	2.7	25.4	9.6	6.7	9753	105.2	424	980.2	368.4	257.4	376,202.6
Chicken	0.9	1.1	9.5	4.5	1310	35.3	142	43.5	364.9	173.5	50,519.1
Egg	0.4	0.5	4.3	1.8	1452	14.6	59	19.6	167.2	67.9	55,988.9
Salmon	0.6	0.4	3.0	10.6	1871	23.6	95	14.6	114.4	408.1	72,157.5
White fish	1.4	0.9	6.8	24.1	4259	53.7	216	33.2	260.4	929.0	164,278.5
Beans, mixed	0.2	1.7	2.4	1.9	2483	7.7	31	66.5	94.2	72.9	95,775.9
Chickpeas	0.2	1.6	2.2	1.7	2270	7.0	28	60.8	86.1	66.6	87,556.9
Lentils	0.2	2.1	2.9	2.3	2988	9.2	37	80.0	113.3	87.7	115,257.0
Baked beans	0.2	2.1	2.9	2.3	2980	9.2	37	79.8	113.0	87.4	114,927.6
Tofu, firm	0.3	0.3	0.6	0.6	479	11.6	47	12.7	24.2	22.4	18,489.7
Tofu, silken	0.7	0.7	1.4	1.3	1074	25.9	104	28.4	54.3	50.2	41,434.5
Mixed nuts	0.0	0.2	0.6	0.3	2598	1.2	5	7.6	23.3	11.4	100,188.6

^#^ Energy content of foods were matched to one serve of beef (471 kJ). ^ Greenhouse gas emissions equivalent to the driving distance by an average passenger vehicle with average fuel economy. Acid., acidifying emissions; Equiv, equivalent; Eutroph., eutrophying emissions; GHG, greenhouse gas. All measures of environmental sustainability were calculated from Poore and Nemecek [13].

**Table 4:** Modeling nutrient intake of pregnant women replacing one serve of beef per week with an isoenergetic serve of firm tofu ^#^.

		Original Intake						Modeled Intake					
Nutrients	NRVs	Mean	SD	Median	IQR	Met NRVs (n)	Met NRVs (%)	Mean	SD	Median	IQR	Met NRVs (n)	Met NRVs (%)
Protein, g	EAR 49	97.8	45.3	87.9	72.2–109.4	112	98	96.5	45.3	86.6	70.9–108.1	112	98
Dietary fiber, g	AI 28	23.3	8.8	22.2	17.6–27.9	27	24	23.7	8.8	22.7	18.1–28.3	29	26
Iron, mg	EAR 22	14.2	6.9	12.7	10.0–16.0	5	5	14.3	6.9	12.9	10.1–16.1	6	5
Zinc, mg	EAR 9.0	12.6	5.0	11.5	9.4–14.2	93	83	12.1	5.0	11.0	8.9–13.7	81	72
Calcium, mg	EAR 840	984.1	332.9	930.8	759.5–1114.7	71	63	1026.2	332.9	972.9	801.6–1156.8	77	69
Dietary Folate equivalents, µg	EAR 520	280.2	118.6	254.6	204.4–323.7	4	4	284.2	118.6	258.7	208.4–327.8	4	4

^#^ Modeling undertaken in 112 pregnant women (65.5%) consuming >1 serve of beef per week. AI, Adequate Intake; EAR, Estimated Average Requirement; NRV, Nutrient Reference Value.

**Table 5:** Modeling the nutrient intake of pregnant women replacing one serve of beef per week with a protein-matched serve of firm tofu ^#^.

		Original Intake						Modeled Intake					
Nutrients	NRVs	Mean	SD	Median	IQR	Met NRVs (n)	Met NRVs (%)	Mean	SD	Median	IQR	Met NRVs (n)	Met NRVs (%)
Dietary fiber, g	AI 28	23.3	8.8	22.2	17.6–27.9	27	24	24.1	8.8	23.0	18.5–28.7	29	26
Iron, mg	EAR 22	14.2	6.9	12.7	10.0–16.0	5	5	14.6	6.9	13.2	10.4–16.4	6	5
Zinc, mg	EAR 9.0	12.6	5.0	11.5	9.4–14.2	93	83	12.3	5.0	11.2	9.1–13.9	87	78
Calcium, mg	EAR 840	984.1	332.9	930.8	759.5–1114.7	71	63	1060.3	332.9	1007.1	835.7–1190.9	82	73
Dietary Folate equivalents, µg	EAR 520	280.2	118.6	254.6	204.4–323.7	4	4	287.4	118.6	261.9	211.6–331.0	4	4

^#^ Modeling undertaken in 112 pregnant women (65.5%) consuming >1 serve of beef per week. AI, Adequate Intake; EAR, Estimated Average Requirement; NRV, Nutrient Reference Value.

## Data Availability

The data presented in this study are available on request from the corresponding author. The data are not publicly available for ethical reasons.

## Supplementary Materials

The following are available online at https://www.mdpi.com/article/10.3390/nu13103355/s1, Figure S1: Flow diagram of participant selection; Table S1: Dietary intake analysis of pregnant women in the Newborn Body Fatness study; Table S2: Food swaps—beef. Differences in nutrients and measures of environmental sustainability between beef and more sustainable isoenergetic options; Table S3: Food swaps—chicken. Differences in nutrients and measures of environmental sustainability between chicken and more environmentally sustainable isoenergetic options; Table S4: Food swaps—white fish. Differences in nutrients and measures of environmental sustainability between white fish and more environmentally sustainable isoenergetic options; Table S5: Food swaps—milk. Differences in nutrients and measures of environmental sustainability between milk and more environmentally sustainable isoenergetic options; Table S6: Nutrient analysis and measures of environmental sustainability of the reference food (beef) and more environmentally sustainable protein-matched options; Table S7: Food swaps—beef. Differences in nutrients and measures of environmental sustainability between beef and more environmentally sustainable protein-matched options; Table S8: Food swaps—chicken. Differences in nutrients and measures of environmental sustainability between chicken and more environmentally sustainable protein-matched options; Table S9: Food swaps—white fish. Differences in nutrients and measures of environmental sustainability between white fish and more environmentally sustainable protein-matched options; Table S10: Food swaps—milk. Differences in nutrients and measures of environmental sustainability between milk and more environmentally sustainable protein-matched options.
